# Esophageal cancer detection via non-contrast CT and deep learning

**DOI:** 10.3389/fmed.2024.1356752

**Published:** 2024-03-06

**Authors:** Chong Lin, Yi Guo, Xu Huang, Shengxiang Rao, Jianjun Zhou

**Affiliations:** ^1^Department of Radiology, Zhongshan Hospital (Xiamen), Fudan University, Shanghai, Fujian, China; ^2^Xiamen Municipal Clinical Research Center for Medical Imaging, Xiamen, Fujian, China; ^3^Departments of Thoracic Surgery, Zhongshan Hospital, Fudan University, Shanghai, China; ^4^Department of Radiology, Zhongshan Hospital, Fudan University, Shanghai, China

**Keywords:** deep learning, no new net, non-contrast chest computed tomography, esophageal cancer, diagnosis

## Abstract

**Background:**

Esophageal cancer is the seventh most frequently diagnosed cancer with a high mortality rate and the sixth leading cause of cancer deaths in the world. Early detection of esophageal cancer is very vital for the patients. Traditionally, contrast computed tomography (CT) was used to detect esophageal carcinomas, but with the development of deep learning (DL) technology, it may now be possible for non-contrast CT to detect esophageal carcinomas. In this study, we aimed to establish a DL-based diagnostic system to stage esophageal cancer from non-contrast chest CT images.

**Methods:**

In this retrospective dual-center study, we included 397 primary esophageal cancer patients with pathologically confirmed non-contrast chest CT images, as well as 250 healthy individuals without esophageal tumors, confirmed through endoscopic examination. The images of these participants were treated as the training data. Additionally, images from 100 esophageal cancer patients and 100 healthy individuals were enrolled for model validation. The esophagus segmentation was performed using the no-new-Net (nnU-Net) model; based on the segmentation result and feature extraction, a decision tree was employed to classify whether cancer is present or not. We compared the diagnostic efficacy of the DL-based method with the performance of radiologists with various levels of experience. Meanwhile, a diagnostic performance comparison of radiologists with and without the aid of the DL-based method was also conducted.

**Results:**

In this study, the DL-based method demonstrated a high level of diagnostic efficacy in the detection of esophageal cancer, with a performance of AUC of 0.890, sensitivity of 0.900, specificity of 0.880, accuracy of 0.882, and F-score of 0.891. Furthermore, the incorporation of the DL-based method resulted in a significant improvement of the AUC values w.r.t. of three radiologists from 0.855/0.820/0.930 to 0.910/0.955/0.965 (*p* = 0.0004/<0.0001/0.0068, with DeLong’s test).

**Conclusion:**

The DL-based method shows a satisfactory performance of sensitivity and specificity for detecting esophageal cancers from non-contrast chest CT images. With the aid of the DL-based method, radiologists can attain better diagnostic workup for esophageal cancer and minimize the chance of missing esophageal cancers in reading the CT scans acquired for health check-up purposes.

## 1 Introduction

Esophageal cancer is the seventh most frequently diagnosed cancer with a high mortality rate and the sixth leading cause of cancer deaths in the world (1–3). The prevalence of esophageal cancer is increasing due to the rising world population, longer longevity, and the popularity of risk factors such as tobacco and alcohol consumption (2, 4, 5). This cancer originates from the inner layer of the esophagus wall and progresses outward, which makes early detection difficult as symptoms are often absent, resulting in late-stage diagnosis and poor prognosis (2, 6). Given its high malignancy and unfavorable outcomes, timely identification is of utmost importance. While endoscopy serves as the gold standard for diagnosing esophageal cancer, its invasiveness and high cost necessitate the exploration of alternative methods to expand the reach of testing (7).

Esophageal carcinomas can manifest in several forms (8). They may appear as a focal area of mural thickening, either with or without ulceration. Another form is a flat or polypoid lesion. Finally, they can also present as generalized mural thickening. According to these characteristics, computed tomography (9) offers opportunities to detect esophageal carcinomas. With the development of medical technology, CT examination is a central modality in modern radiology contributing to diagnostic medicine in almost every medical subspecialty and has become increasingly convenient and common (10). Traditionally, contrast CT was used to detect esophageal carcinomas (8), but with the development of deep learning (DL) technology, it may now be possible for CT to detect early-stage esophageal carcinomas.

DL (6) is a type of representation learning method with complex multi-layer neural network architecture and has emerged as the state-of-the-art machine learning method in many applications (11, 12). In radiology, DL techniques have the most significant impact: lesion or disease detection (13–15), classification (16, 17), quantification, and segmentation (12, 17, 18). Examples of these applications include the identification of pulmonary nodules (19, 20) and breast cancer (21), classification of benign or malignant lung nodules (22) and breast tumors (23), utilization of texture-based radiomic features for predicting therapy response in gastrointestinal cancer (24), and segmentation of brain anatomy (25, 26).

The applications of DL methods are gradually common. However, the early detection of esophageal cancer with DL methods is relatively limited. On the other hand, since the esophagus is a hollow organ with contractile and diastolic functions, there are still several challenges in the clinical early diagnosis of esophageal cancer. The benefits and disadvantages of CT with DL to detect esophageal carcinomas are worth exploring.

In this study, we aimed to establish a DL-based diagnostic system to detect esophageal cancer from non-contrast chest CT images. There were 397 esophageal cancer patients and 250 healthy individuals enrolled to train the model. Then, 100 esophageal cancer patients and 100 healthy individuals were included for validation. We compared the diagnostic efficacy of the DL model with that of radiologists at different expertise levels, both with and without the reference to the DL model.

## 2 Materials and methods

### 2.1 Data sets

This retrospective dual-center study included non-contrast chest CT images of 397 primary esophageal cancer patients and 250 healthy individuals, collected from July 2017 to December 2022 at Zhongshan Hospital (Xiamen), for the purpose of training the model, then 100 esophageal cancer patients and 100 healthy individuals were enrolled from October 2015 to August 2019 at Zhongshan Hospital for validation (Table 1). The inclusion criteria of esophageal cancer patients were as follows: patients with pathologically proven esophageal cancer through endoscopic biopsy or surgical pathology with non-contrast chest CT images from the thoracic inlet to the esophagogastric junction and patients who had no other disease that could cause thickening of the esophageal wall, such as varicocele caused by liver cirrhosis. Non-esophageal cancer subjects were enrolled randomly from the health checkup centers and were imaged with chest CT scans. These subjects were confirmed to be negative for esophageal cancer in the following 2 years. Patients were excluded from the dataset if any clinical data was incomplete, or the quality of chest CT scans was poor.

**Table 1:** Patient background information.

	Esophageal cancer negative		Esophageal cancer positive	
	Training (*n* = 250)	Validation (*n* = 100)	Training (*n* = 397)	Validation (*n* = 100)
Ages (years)	54.79 ± 12.42	58.475 ± 9.83	64.86 ± 9.58	61.91 ± 7.91
Female/Male	100/150	45/55	93/304	10/90
Main location	N/A	N/A		
Upper thoracic	N/A	N/A	34	12
Middle thoracic	N/A	N/A	209	63
Lower thoracic	N/A	N/A	154	25
Length of esophageal cancers (mm)	N/A	N/A	44.92 ± 22.26	25.41 ± 12.08
T stage	N/A	N/A		
T_1_	N/A	N/A	18	5
T_2_	N/A	N/A	63	15
T_3_	N/A	N/A	185	53
T_4_	N/A	N/A	26	26
T_x_	N/A	N/A	105	1
Pathology	N/A	N/A		
SCC	N/A	N/A	349	95
Adenocarcinoma	N/A	N/A	37	2
Other	N/A	N/A	11	3

### 2.2 Computed tomography image acquisition

All images were scanned by Revolution CT, GE Discovery CT750 HD, 512-slice LightSpeed VCT (GE Medical Systems), Aquilian one (Canon Medical Systems Corporation), and uCT 760, 128-slice (United imaging) with parameter setting: tube voltage as 120 kVp, tube current as 100 ~ 750 mA, image slice matrix as 512 × 512, and slice thickness as 5 mm.

### 2.3 CT-image convolutional neural network

The nnU-Net is a powerful neural network specifically designed for medical image segmentation. The nnU-Net is based on 2D and 3D U-Net models geared with several technical improvements (27). For instance, in terms of preprocessing and post-processing, the nnU-Net applies various methods such as denoising, enhancement, cropping, thresholding, and fusion to improve image quality and segmentation results, while also enhancing the visualization and interpretability of segmentation outcomes. For model optimization, the nnU-Net employs an optimizer with adaptive learning rate and momentum to expedite the training process and enhance the performance of the model. In model training, the cross-validation scheme is implemented for the selection of the best-performing model. These technical improvements promise that the nnU-Net can yield more robust models.

In previous research, the nnU-Net has been widely used for the segmentation of the aorta (28), carotid artery (29), liver (30), and fetal brain (31), with promising performance in terms of accuracy, reliability, and efficiency. Accordingly, the nnU-Net is employed for the segmentation of the esophagus in the CT images with the evaluation metrics of Dice coefficient and Hausdorff Distance.

**Figure 1:**
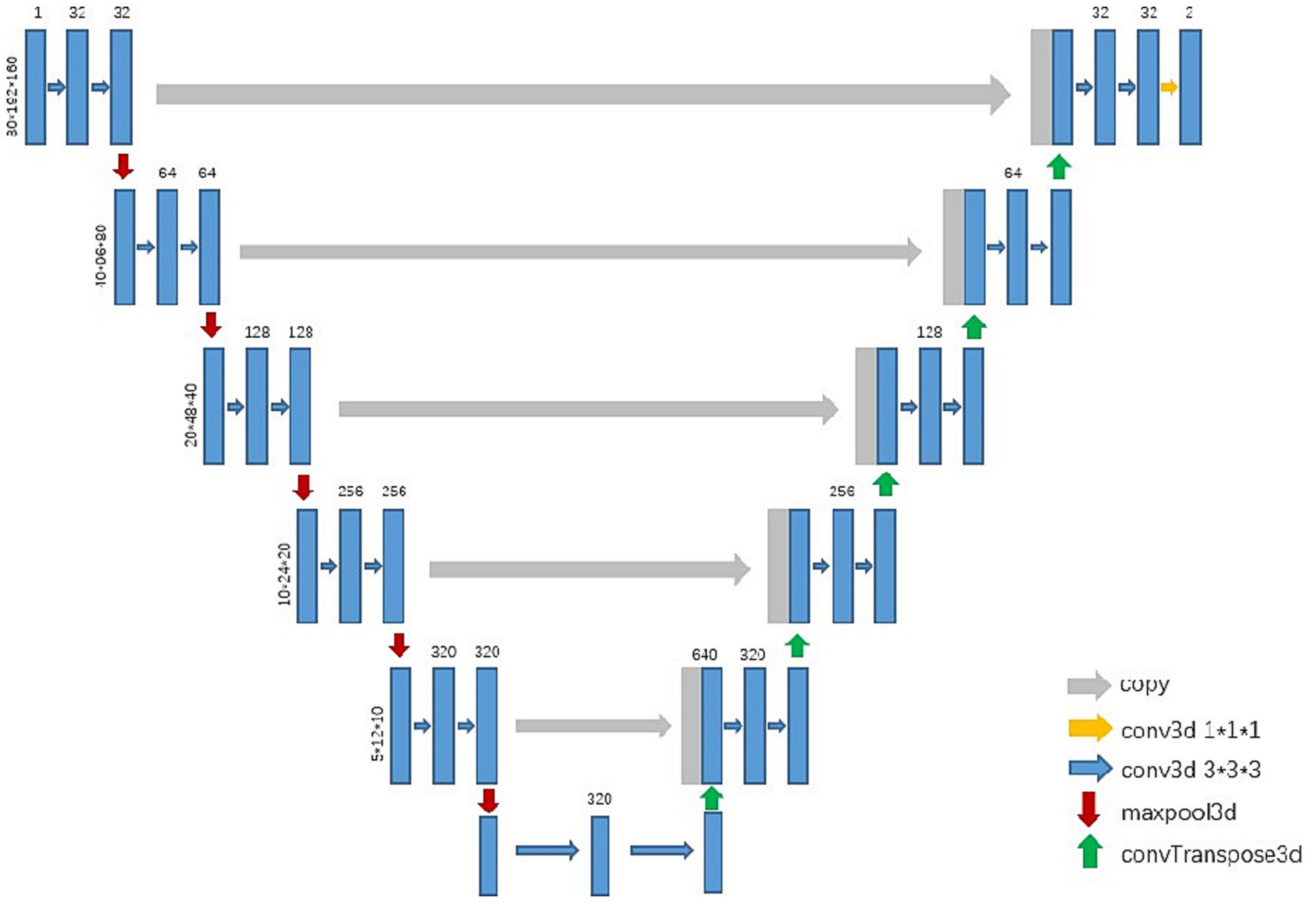
Flow diagram of the nnU-net.

In the experiment, we trained a 3d U-Net model to segment the esophagus (see Figure 1). After preprocessing the training data, the networks automatically cropped the image patch with the sizes 80, 192, and 160 for training. The initial learning rate was 0.01, which continuously decreased with the increase in the number of iterations, and it no longer decreased when it reached 0.001. The networks were optimized with SGD and the training loss was dice loss.

**Figure 2:**
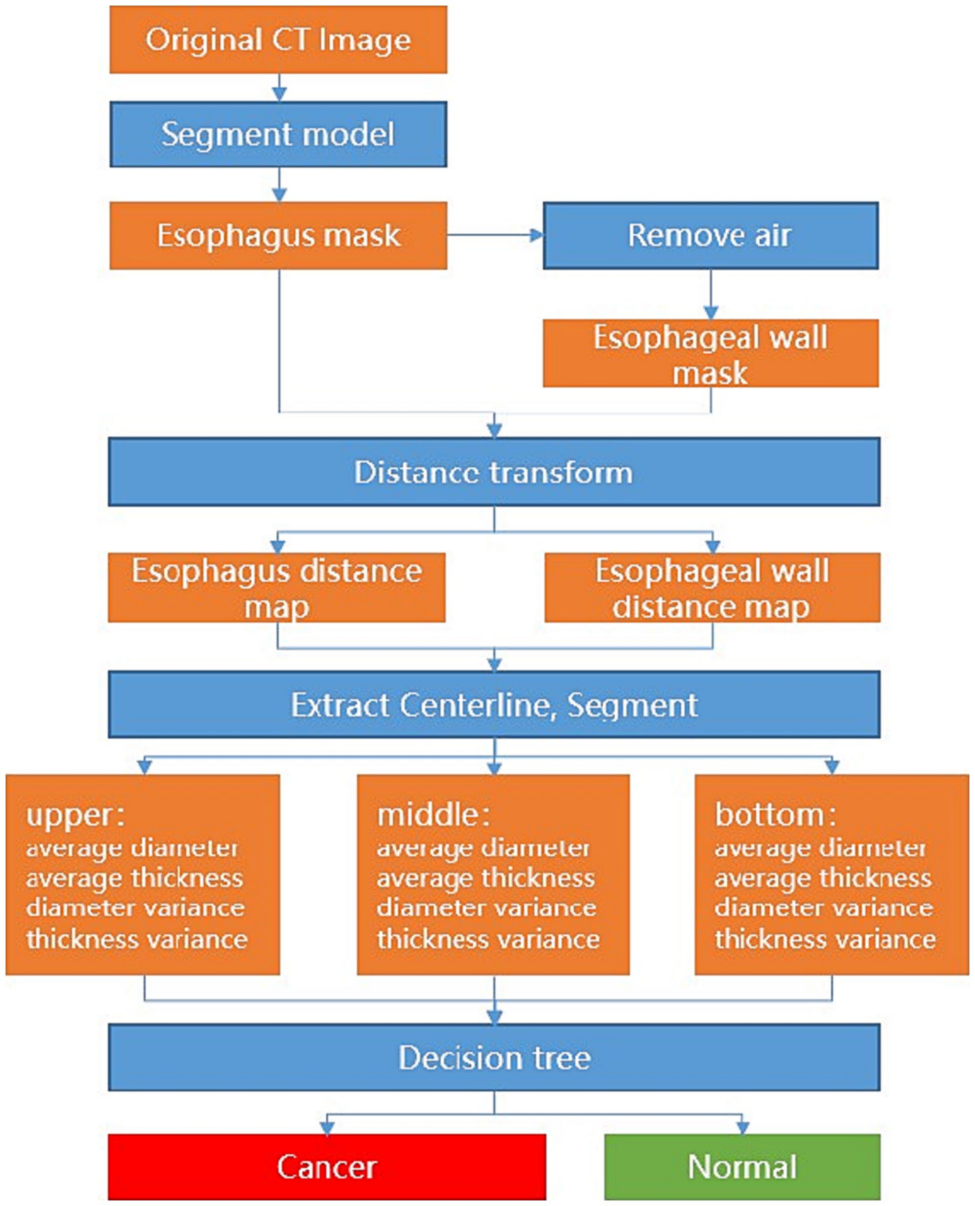
Flow diagram of the deep learning model.

Specifically, the nnU-Net demarcates the esophagus, and a post-processing of the appropriate thresholding for the removal of the air portions within the esophagus is applied to delineate the esophageal wall. Afterward, the average diameter and wall thickness of the esophagus can be calculated through distance transform, see Figure 1. In clinical definition, the esophagus is typically divided into upper, middle, and lower segments. In such cases, each segment may need different analytical methods and treatments. To mimic the clinical analytical paradigm, the center line is computed from the esophagus and further straightened to facilitate the automatic division of the upper, middle, and lower segments with intervals of 5 cm, 10 cm, and the remaining length from the starting point, respectively. For each segment, the measurement variances of the diameter and wall thickness of sampled transversal cut-planes are further computed. With these measurement variances, a decision tree is applied to determine if esophageal cancer is presented in the corresponding segment, see Figure 2.

### 2.4 The clinical application of the DL model

To assess the efficacy of the model in clinical application for the detection of esophageal cancer, three radiologists participated in this study. The participants reviewed the CT images in the validation dataset independently, which were presented in a randomized sequence, and made diagnoses either on their own or with the assistance of the model. The detailed reading protocol is elaborated as follows.

Two junior radiologists, Radiologist 1 and Radiologist 2, with 5 years of image diagnosis experience, and one senior radiologist, Radiologist 3, with 13 years of experience were invited to this study. All three radiologists were involved in the reading and diagnosis of the validation set tests. None of them had any knowledge of the study’s purpose or any clinical information. Each radiologist independently reviewed the CT images of the validation dataset and made routine diagnostic practices. The diagnostic efficiency of each radiologist, including sensitivity, specificity, accuracy, F1 score, and AUC, was then calculated.

**Figure 3:**
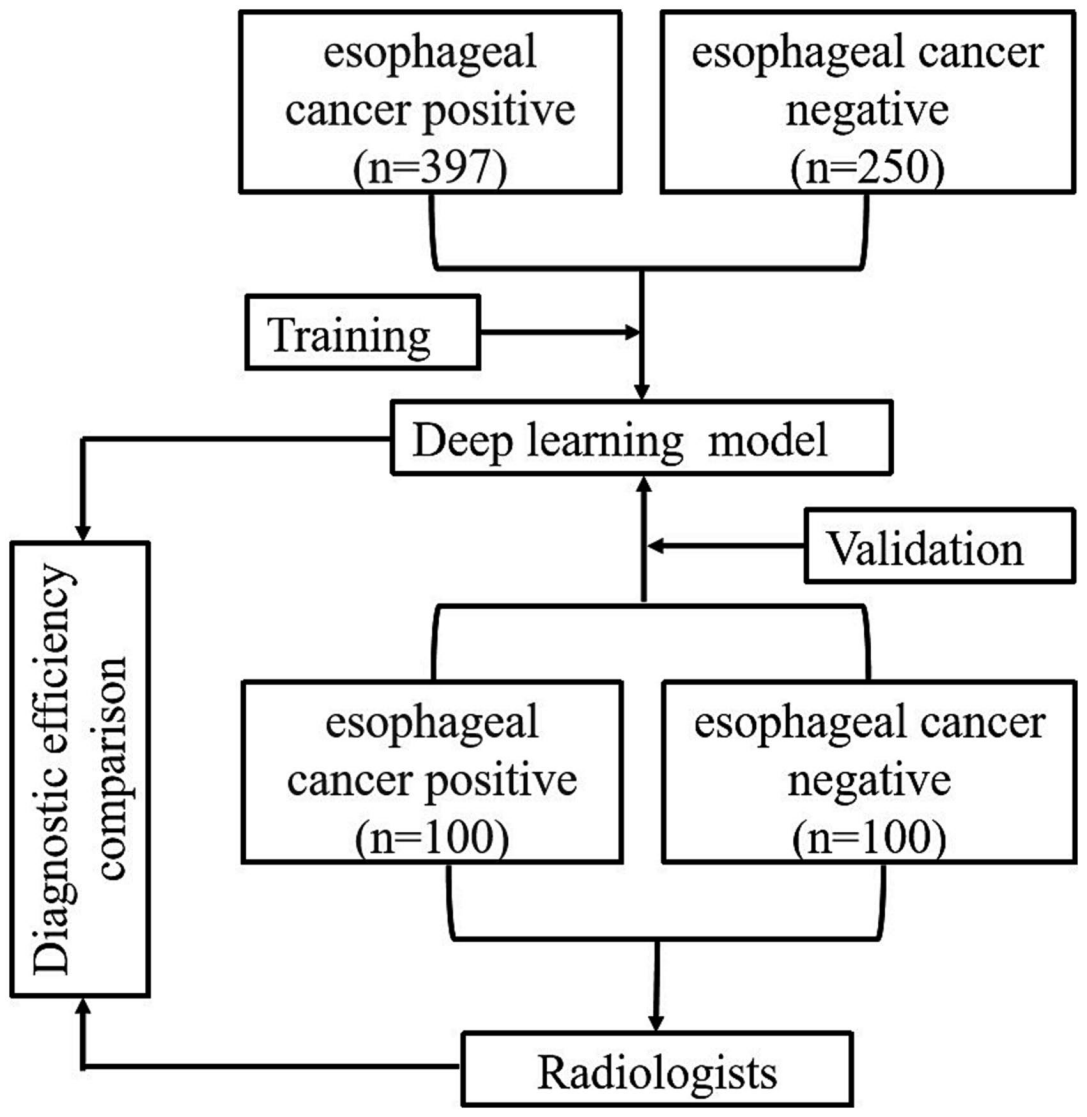
Experimental flow chart of the study.

After a 3-month memory washout period, the three radiologists reevaluated the CT images of the validation dataset with the assistance of the DL model and made another round of diagnoses. The diagnostic workups of each radiologist, with the aid of the model, were further assessed with the same evaluation metrics. Finally, a quantitative comparison was performed to illustrate the diagnostic efficacy among the image diagnostic workups of radiologists, with and without the assistance of the DL model, as well as the pure prediction results from the DL model. The total flow diagram of the study is shown in Figure 3.

### 2.5 Statistical analysis

In the classic evaluation paradigm for a classification model, four basic metrics of true positive (TP), true negative (TN), false negative (FN), and false positive (FP) may commonly need to be calculated for the computation of sensitivity and specificity. In this study, a TP suggests true cancer identification, whereas TN is the true non-cancerous classification. The FN indicates a missing cancer finding by either the model or the radiologist, while the FP represents a false cancer finding from the radiologist or DL model. In addition to sensitivity and specificity, the metrics of precision, false negative rate (FNR), false positive rate (FPR), and F1 score are computed to support extensive and quantitative performance comparison. The mentioned evaluation metrics are defined as follows.


(1) $$\mathrm{Sensitivity}=TP/\left(TP+FN\right)\text{,}$$

(2) $$\mathrm{Specificity}=TN/\left(TN+FP\right)\text{,}$$

(3) $$\mathrm{Recall}=TP/\left(TP+FN\right)\text{,}$$

(4) $$\mathrm{Precision}=TP/\left(TP+FP\right)\text{,}$$

(5) FNR=1−sensitivity,

(6) FPR=1−specificity,

(7) $$F1\;\mathrm{score}=2*\left(\mathrm{Recall}*\mathrm{Precision}\right)/\left(\mathrm{Recall}+\mathrm{Precision}\right)\text{.}$$

Meanwhile, the area under the receiver operating characteristic (ROC) curve (AUC) was also employed as another quantitative metric (32). We used the intraclass correlation efficient (ICC) to compare the diagnosis consistency between the two junior radiologists. The ICC (95%CI) was 0.942 (0.924 and 0.955), which showed good diagnosis consistency. To further compare the performance of the DL model as well as the readers’ performance with and without the referencing of the DL model, DeLong’s test for AUC was adopted (33). The overall statistical analyses were carried out with software packages of SPSS 26.0 and MedCalc 22.016. Continuous variables were presented as mean ± standard deviation. Statistical significance was defined at a value of p of less than 0.05.

## 3 Results

In this study, CT scans of 397 primary esophageal cancer patients and 250 healthy individuals were involved in training the DL model, whereas independent images of 100 esophageal cancer patients and 100 healthy individuals were used for validation. Table 1 summarizes the background of all 497 primary esophageal cancer patients and 350 healthy individuals.

### 3.1 The diagnostic efficiency of the DL model in the validation data set

The nnU-Net-based DL model was evaluated in a five-fold cross-validation scheme. The DICE for the esophagus segmentation in the validation data set was 0.875 ± 0.0728 and Hausdorff Distance was 1.765 ± 0.154. The performance of the DL model in the validation data set was summarized in Table 2. In the validation data set, the AUC of the model was 0.890, whereas the metrics of sensitivity, specificity, accuracy, and F1 score were 0.900, 0.880, 0.882, and 0.891, respectively.

**Table 2:** Diagnostic efficiency comparison between deep learning model and radiologists.

	Sensitivity	Specificity	Accuracy	F1 score	AUC	FNR	FPR	P^a^	P^b^
Deep learning model	0.900	0.880	0.882	0.891	0.890	0.100	0.120		
Radiologists 1 independently	0.860	0.850	0.855	0.856	0.855	0.140	0.150	0.0040	
Radiologists 2 independently	0.780	0.870	0.857	0.817	0.820	0.220	0.130	0.0001	
Radiologists 3 independently	0.950	0.910	0.913	0.931	0.930	0.050	0.090	0.0040	
Radiologists average 1	0.860	0.877	0.873	0.866	0.867	0.137	0.123		
Radiologists 1 with model	0.920	0.900	0.902	0.911	0.910	0.080	0.100	0.0444	0.0004
Radiologists 2 with model	0.960	0.950	0.950	0.955	0.955	0.040	0.050	0.0002	<0.0001
Radiologists 3 with model	0.960	0.970	0.970	0.965	0.965	0.040	0.030	0.0001	0.0068
Radiologists average 2	0.947	0.940	0.941	0.944	0.943	0.053	0.060		

P^a^: Comparisons of AUC value between the deep learning model and each radiologist with or without the deep learning model.

P^b^: Comparisons of AUC value between the radiologist with and without the deep learning model.

Among the 10 CT examinations segmented by all three radiologists, the segmentations created by the different radiologists were shown to be similar. As shown in Table 3, Median interreader DSC ranged from 0.80 to 0.89 for all CT examinations. Median model-reader DSC ranged from 0.76 to 0.88 for all scans. The interreader DSC was not different than the model-reader DSC, indicating that the segmentation performance of the machine-learning algorithm did not differ significantly from that of the radiologists.

**Table 3:** Median interreader and radiologists-model DSCs for 10 cases in the test set.

Reader no.	Radiologists 1	Radiologists 2	Radiologists 3	Model
Radiologists 1	1	0.80 (0.65–0.96)	0.89 (0.79–0.98)	0.87 (0.73–0.99)
Radiologists 2		1	0.81 (0.68–0.88)	0.76 (0.64–0.83)
Radiologists 3			1	0.87 (0.76–0.98)

Data are Dice similarity coefficients (DSCs), with minimum and maximum values in parentheses.

### 3.2 The diagnostic efficiency of radiologists with and without referring to the results of the DL model

**Figure 4:**
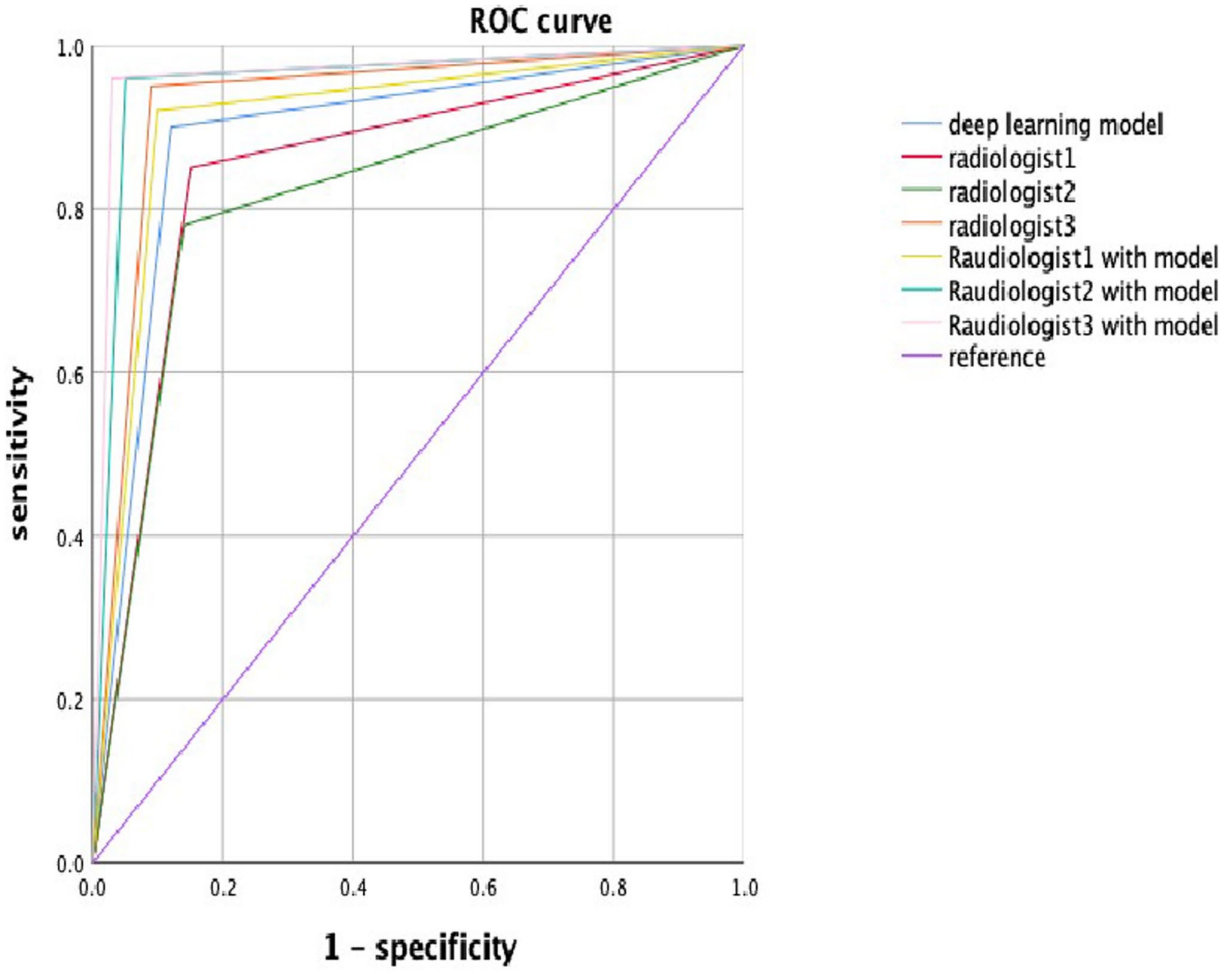
The ROC curve in the deep learning model and radiologists with or without the deep learning model. Blue, red, green, orange, lemon-yellow, blue-green, and pink lines indicate the ROC curve of the deep learning model, radiologist1, radiologist2, radiologist3, radiologist1 with the model, radiologist2 with the model, and radiologist3 with the model.

The diagnostic efficiency of the radiologists in the validation data set is shown in Table 2. The AUC of Radiologist 1 independently in the validation set was 0.855, whereas the metrics of sensitivity Equation (1), specificity Equation (2), accuracy, and F1 score Equations (3, 4, 7) were 0.860, 0.850, 0.855, and 0.856, respectively. The AUC of Radiologist 2 independently in the validation set was 0.820, with the sensitivity, specificity, and F1 score of 0.780, 0.870, and 0.817, respectively. The AUC of Radiologist 3 independently in the validation set was 0.930, with the sensitivity, specificity, and F1 score of 0.950, 0.910, and 0.931, respectively. The diagnostic performance of the DL model is better than Radiologist 1 and Radiologist 2 independently with statistical significance in the AUC; however, it was lower than Radiologist 3 significantly. The other metrics of sensitivity, specificity, and F1 score were also attained higher by the DL model than Radiologist 1 and Radiologist 2, but lower than Radiologist 3. With the help of the model, Radiologist 1 and Radiologist 2 showed significant improvement in the AUC, as well as the other metrics. Meanwhile, the performance of Radiologist 3 also improved with the DL model when compared to the performance in the independent reading session. Figure 4 visually compares the ROC curves of the DL model and the radiologists.

### 3.3 Comparison of the rates of misdiagnosis and missed diagnosis between DL model and radiologists

In the validation set, the DL model missed 10% of esophageal cancer cases [FNR = 0.100, Equation (5)], which was lower than the average FNR of 13.7% for all radiologists in the independent reading session (without the DL model). With the incorporation of DL modeling in the reading session, the average FNR by all radiologists was lowered to 5%. In such cases, the DL model can improve radiologists’ workups in finding esophageal cancers. On the other hand, the DL model yielded 12% false positives in the validation set, which was similar to the average FPR Equation (6) of 12.3% by all radiologists in independent reading sessions. With the aid of the DL model, the average FPR by all radiologists was reduced to 6%, see Table 2. Accordingly, the DL model can on average improve radiologists’ performance and reduce the FP and FN rates in half.

**Figure 5:**
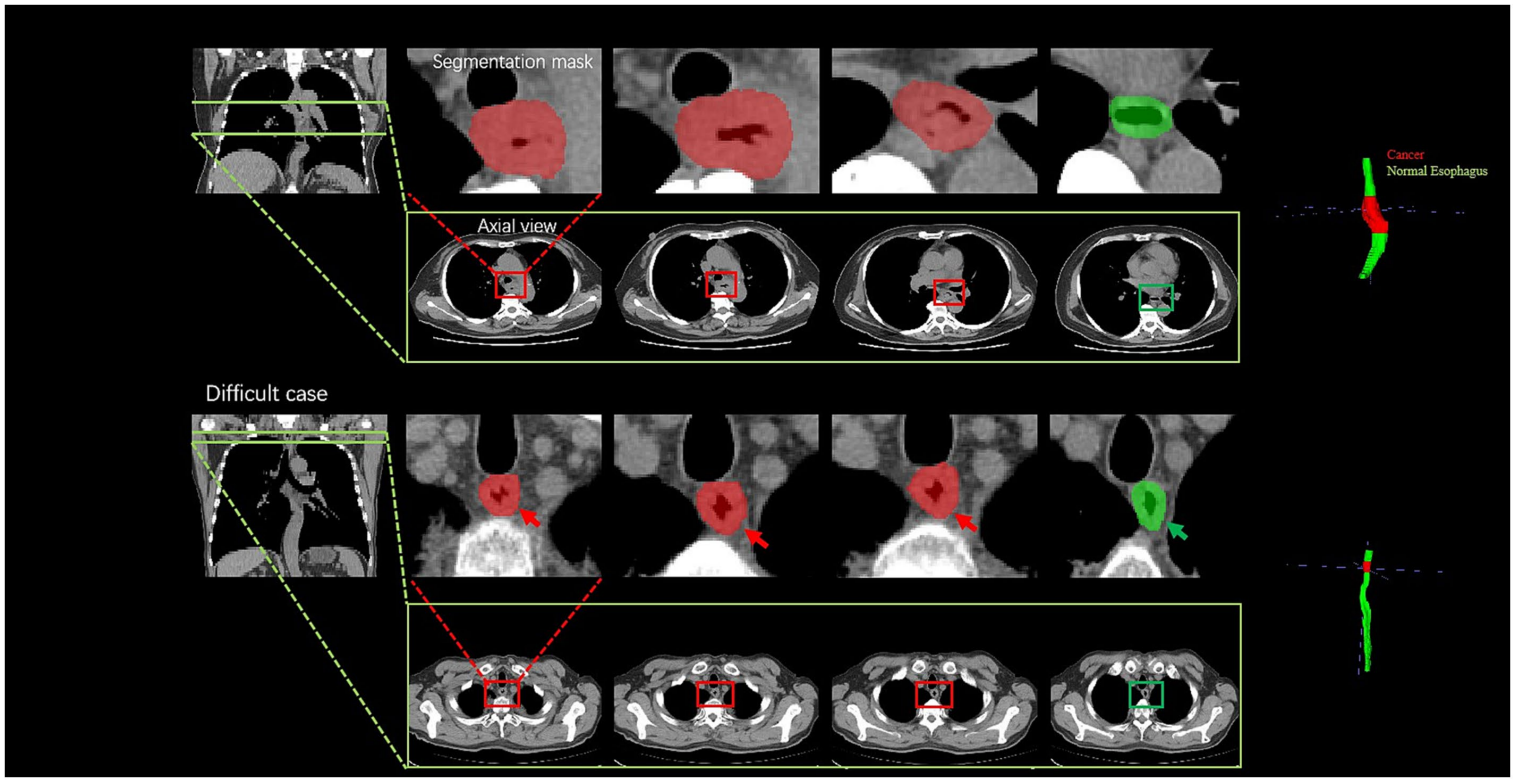
Visualization of two cancer cases. For the easy case, there is a significant thickening of the diameter and thickening of the esophageal wall; the difficult case is a 77-year-old man diagnosed with T1 stage esophageal cancer; the radiologists failed to accurately diagnose the cancer, whereas the deep learning model successfully detected it by the subtle variation of the esophageal wall thickness. The cancer part is indicated by the red color, and the green color part presents a normal esophagus.

**Figure 6:**
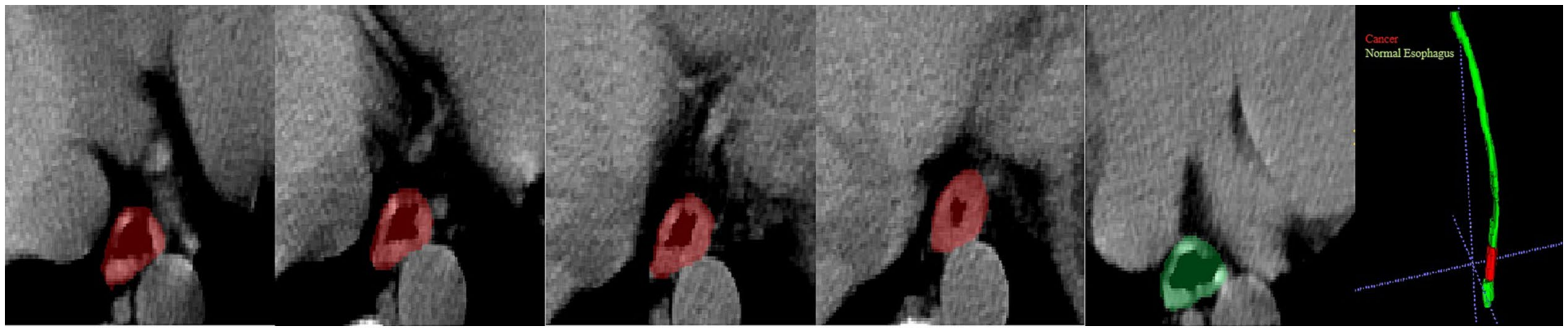
Visualization of a missed diagnosed case by DL. A 62-year-old man diagnosed with T1 stage esophageal cancer under an endoscope; the pathology showed the cancer was confined to the lamina propria of the mucosa and very close to the cardia. The cancer part is indicated by the red color, and the green color part presents a normal esophagus.

Further analysis was conducted for the FPs yielded by the DL model. The majority of FPs were acute and chronic esophagitis (75%, nine cases), and a small proportion were esophageal papillomas, esophageal hyperplastic polyps, and gastric mucosal ectopies (25%, one case for each abnormality). For the FN cases by the DL model, most of them were early-stage cancers, involving seven cases (70%) of esophageal cancer at T1-2 and three cases (30%) of T3-4 esophageal cancer. The DL model missing the T3-4 cancers may be because the nearby soft tissues around the cancers are complicated which further confused the model to an incorrect differentiation. Additionally, a challenging case involving a 77-year-old man diagnosed with T1 stage esophageal cancer was missed by the radiologists but successfully detected by the DL model (Figure 5), which revealed the excellent performance of the DL model. There were still some cases that were too early and did not have detectable changes in the images to be detected, see Figure 6.

## 4 Discussion

In this retrospective dual-center study, a DL-based method was developed to detect esophageal cancer to assist the clinical reading. The model was trained with non-contrast chest CT scans acquired from 397 esophageal cancer-positive patients and 250 individuals with no esophageal cancer. In the validation, the DL-based method showed a satisfactory diagnostic efficacy in detecting esophageal cancer with an AUC of 0.890 and an accuracy of 0.882, which were higher than the two junior radiologists, i.e., Radiologist 1 and Radiologist 2, but lower than the senior radiologist (Radiologist 3). Referring to the previous study, the underlying reasons the DL model outperformed the junior radiologists may be two-fold (34). First, the DL model was trained by the esophageal cancer cases which were validated by pathology. The junior radiologists did not get systematic and sufficient training in the reading and diagnosis of esophageal cancer in *non-contrast chest* CT images. Second, DL algorithms have a higher sensitivity to subtle image changes than human eyes, and hence yield better detection results for the easy-missing lesions like esophageal cancers (32). With the help of the DL model, the junior and senior radiologists achieved better diagnostic workups in detecting esophageal cancers. Accordingly, the computerized DL system may be potentially valuable in the context of health checkup non-contrast CT examination for the early detection of esophageal cancers.

In this study, our model reached the performance of sensitivity, specificity, accuracy, F1 score, and AUC values of 0.900, 0.880, 0.882, 0.891, and 0.890, respectively, which was better than the previous study with V-net, where the sensitivity, specificity, and AUC were 0.690, 0.610, and 0.650, respectively (35). It may be because our method is equipped with a more robust segmentation model and better cancer identification post-processing scheme for better results. Compared to the method with the pure image classification model of VGG16 on the *contrast-enhanced CT*, the reported performance of sensitivity, specificity, accuracy, and F1 score were 0.717, 0.90, 0.842, and 0.742, respectively (36). Accordingly, a segmentation model may be helpful to improve the detection performance with slightly lower specificity. On the other hand, another image classification CNN for the *contrast-enhanced chest images* suggested a performance with metrics of sensitivity, specificity, accuracy, and AUC as 0.87, 0.92, 0.92, and 0.95 (33), respectively, since the contrast-enhanced CT may better depict the esophageal cancers and may ease the algorithmic difficulty for DL models. However, our experimental results suggested that the DL can also assist radiologists in improving the workups of esophageal cancers by reducing FPs and FNs in non-contrast chest CT scans. In particular, the DL model may improve the performance of junior radiologists to the senior level, which resonates with the conclusion of the studies (33, 35). Accordingly, this may shed light on the early detection of esophageal cancers, especially in the context of health check-up examinations.

There are several limitations in our study. First, the distribution of sex and age were uneven in the training and validation data, but the esophageal cancers mainly occurred in men and ages >50 years (9); therefore, the enrolled individuals were suitable for model training. Second, we enrolled some early-stage esophageal cancer in this study. However, the DL model and the radiologists failed to identify all these cases. The detection of early-stage esophageal cancer can be very challenging for both the radiologist and the DL model (such as Figure 6), but it is important for clinical practice. Referencing the other studies (33, 36), the contrast-enhanced CT images may provide more information about esophageal cancer from early to late stage than the non-contrast images. Accordingly, we will consider incorporating contrast-enhanced CT to augment the capability of the DL model. Third, for some patients with neoadjuvant chemotherapy before the surgical operation, we obtained the pathology from endoscopic biopsy and did not get the true cancer stage. Fourth, this study involved a medium number of patients. A further expansion of the cohort is needed.

## 5 Summary statement

The DL model can detect esophageal cancer from non-contrast chest images with good sensitivity and specificity. With the help of the DL model, the radiologist can improve the diagnostic efficacy in detecting esophageal csancer, shorten the training time for junior radiologists, and reduce the missed diagnosis of esophageal cancer in routine physical examinations of individuals with only non-contrast chest CT images.

## Data availability statement

The original contributions presented in the study are included in the article/Supplementary material, further inquiries can be directed to the corresponding authors.

## Ethics statement

The studies involving humans were approved by the Ethics Committee of Fudan University Zhongshan Hospital (Xiamen). The studies were conducted in accordance with the local legislation and institutional requirements. Written informed consent for participation was not required from the participants or the participants’ legal guardians/next of kin in accordance with the national legislation and institutional requirements.

## Author contributions

CL: Data curation, Formal analysis, Investigation, Software, Writing – original draft. YG: Data curation, Formal analysis, Investigation, Methodology, Software, Writing – original draft, Validation. XH: Data curation, Writing – original draft. SR: Supervision, Writing – original draft, Writing – review & editing. JZ: Supervision, Writing – original draft, Writing – review & editings.

## References

[1] Uhlenhopp DJ Then EO Sunkara T Gaduputi V. Epidemiology of esophageal. cancer: update in global trends, etiology and risk factors. Clin J Gastroenterol. (2020) 13:1010–21. doi: 10.1007/s12328-020-01237-x, PMID: 32965635

[2] Liu CQ Ma YL Qin Q Wang PH Luo Y Xu PF. Epidemiology of. Esophageal cancer in 2020 and projections to 2030 and 2040. Thorac. Cancer. (2023) 14:3–11. doi: 10.1111/1759-7714.14745 PMC9807450 36482832

[3] Sung H Ferlay J Siegel RL Laversanne M Soerjomataram I Jemal A. Global Cancer statistics 2020: GLOBOCAN estimates of incidence and mortality worldwide for 36 cancers in 185 countries. CA Cancer J Clin. (2021) 71:209–49. doi: 10.3322/caac.21660, PMID: 33538338

[4] Huang FL Yu SJ. Esophageal cancer: risk factors, genetic association, and treatment. Asian J Surg. (2018) 41:210–5. doi: 10.1016/j.asjsur.2016.10.005 27986415

[5] Kamangar F Nasrollahzadeh D Safiri S Sepanlou SG Fitzmaurice C Ikuta KS. The global, regional, and national burden of oesophageal cancer and its attributable risk factors in 195 countries and territories, 1990-2017: a systematic analysis for the global burden of disease study 2017. Lancet Gastroenterol Hepatol. (2020) 5:582–97. doi: 10.1016/S2468-1253(20)30007-8, PMID: 32246941 PMC7232026

[6] Wei MT Friedland S. Early esophageal Cancer what the gastroenterologist needs to know. Gastroenterol Clin N Am. (2021) 50:791–08. doi: 10.1016/j.gtc.2021.07.004 34717871

[7] Smyth EC Lagergren J Fitzgerald RC Lordick F Shah MA Lagergren P. Oesophageal cancer. Nat Rev Dis Primers. (2017) 3:3. doi: 10.1038/nrdp.2017.48 PMC6168059 28748917

[8] Ba-Ssalamah A Zacherl J Noebauer-Huhmann IM Uffmann M Matzek WK Pinker K. Dedicated multi-detector CT of the esophagus: spectrum of diseases. Abdom Imaging. (2009) 34:3–18. doi: 10.1007/s00261-007-9290-5, PMID: 17653787

[9] Obermannova R Alsina M Cervantes A Leong T Lordick F Nilsson M. Oesophageal cancer: ESMO clinical practice guideline for diagnosis, treatment and follow-up. Ann Oncol. (2022) 33:992–04. doi: 10.1016/j.annonc.2022.07.003 35914638

[10] Arndt C Guttler F Heinrich A Burckenmeyer F Diamantis I Teichgraber U. Deep Learning CT image reconstruction in clinical practice. Rofo. (2021) 193:252–61. doi: 10.1055/a-1248-2556, PMID: 33302311

[11] LeCun Y Bengio Y Hinton G. Deep learning. Nature. (2015) 521:436–44. doi: 10.1038/nature14539 26017442

[12] McBee MP Awan OA Colucci AT Ghobadi CW Kadom N Kansagra AP. Deep Learning in radiology. Acad Radiol. (2018) 25:1472–80. doi: 10.1016/j.acra.2018.02.018 29606338

[13] Wang QF Zhou XH Wang C Liu ZQ Huang J Zhou Y. WGAN-based synthetic minority over-sampling technique: improving semantic fine-grained classification for lung nodules in CT images. IEEE Access. (2019) 7:18450–63. doi: 10.1109/ACCESS.2019.2896409

[14] Ouyang X Karanam S Wu ZY Chen TC Huo JY Zhou XS. Learning hierarchical attention for weakly-supervised chest X-ray abnormality localization and diagnosis. IEEE Trans Med Imaging. (2021) 40:2698–10. doi: 10.1109/TMI.2020.3042773, PMID: 33284748

[15] Li ZR Cui ZM Wang S Qi YJ Ouyang X Chen QT. Domain generalization for mammography detection via multi-style and multi-view contrastive Learning. Medical image computing and computer assisted intervention - Miccai 2021. Electr Eng Sys Sci. (2021) 12907:98–108. doi: 10.1007/978-3-030-87234-2_10

[16] Cheng JZ Ni D Chou YH Qin J Tiu CM Chang YC. Computer-aided. Diagnosis with Deep Learning architecture: applications to breast lesions in US images and pulmonary nodules in CT scans. Sci Rep. (2016) 6:24454. doi: 10.1038/srep24454 27079888 PMC4832199

[17] Sihong C Jing Q Xing J Baiying L Tianfu W Dong N. Automatic scoring of multiple semantic attributes with multi-task feature leverage: a study on pulmonary nodules in CT images. IEEE Trans Med Imaging. (2017) 36:802–14. doi: 10.1109/TMI.2016.2629462 28113928

[18] Lei BY Huang S Li H Li R Bian C Chou YH. Self-co-attention neural. Network for anatomy segmentation in whole breast ultrasound. Med Image Anal. (2020) 64:101753. doi: 10.1016/j.media.2020.101753 32574986

[19] Wang C Elazab A Wu J Hu Q. Lung nodule classification using deep feature. Fusion in chest radiography. Comput Med Imaging Graph. (2017) 57:10–8. doi: 10.1016/j.compmedimag.2016.11.004, PMID: 27986379

[20] Saihood A Karshenas H Nilchi ARN. Deep fusion of gray level co-occurrence. Matrices for lung nodule classification. PLoS One. (2022) 17:e0274516. doi: 10.1371/journal.pone.0274516, PMID: 36174073 PMC9521911

[21] Chae EY Kim HH Jeong JW Chae SH Lee S Choi YW. Decrease in. Interpretation time for both novice and experienced readers using a concurrent computer-aided detection system for digital breast tomosynthesis. Eur Radiol. (2019) 29:2518–25. doi: 10.1007/s00330-018-5886-0, PMID: 30547203

[22] Ciompi F Chung K van Riel SJ Setio AAA Gerke PK Jacobs C. Towards. Automatic pulmonary nodule management in lung cancer screening with deep learning. Sci Rep. (2017) 7:46479. doi: 10.1038/srep46479, PMID: 28422152 PMC5395959

[23] Thirumalaisamy S Thangavilou K Rajadurai H Saidani O Alturki N Mathivanan SK. Breast Cancer classification using synthesized Deep Learning model with metaheuristic optimization algorithm. Diagnostics (Basel). (2023) 13:2925. doi: 10.3390/diagnostics13182925 PMC10528264 37761292

[24] Wong PK Chan IN Yan HM Gao S Wong CH Yan T. Deep learning. Based radiomics for gastrointestinal cancer diagnosis and treatment: a minireview. World J Gastroenterol. (2022) 28:6363–79. doi: 10.3748/wjg.v28.i45.6363 36533112 PMC9753055

[25] Akkus Z Galimzianova A Hoogi A Rubin DL Erickson BJ. Deep Learning for. Brain MRI segmentation: state of the art and future directions. J Digit Imaging. (2017) 30:449–59. doi: 10.1007/s10278-017-9983-4, PMID: 28577131 PMC5537095

[26] Yamanakkanavar N Choi JY Lee B. MRI segmentation and classification of. Human brain using Deep Learning for diagnosis of Alzheimer's disease: a survey. Sensors (Basel). (2020) 20:3243. doi: 10.3390/s20113243, PMID: 32517304 PMC7313699

[27] Isensee F Jaeger PF Kohl SAA Petersen J Maier-Hein KH. nnU-Net: a self-configuring method for deep learning-based biomedical image segmentation. Nat Methods. (2021) 18:203–11. doi: 10.1038/s41592-020-01008-z 33288961

[28] Li F Sun L Lam KY Zhang S Sun Z Peng B. Segmentation of human. Aorta using 3D nnU-net-oriented deep learning. Rev Sci Instrum. (2022) 93:114103. doi: 10.1063/5.0084433, PMID: 36461517

[29] Zhu Y Chen L Lu W Gong Y Wang X. The application of the nnU-Net-based. Automatic segmentation model in assisting carotid artery stenosis and carotid atherosclerotic plaque evaluation. Front Physiol. (2022) 13:1057800. doi: 10.3389/fphys.2022.1057800, PMID: 36561211 PMC9763590

[30] Pettit RW Marlatt BB Corr SJ Havelka J Rana A nnU-Net Deep Learning. Method for segmenting parenchyma and determining liver volume from computed tomography images. Ann Surg Open. (2022) 3:e155. doi: 10.1097/AS9.0000000000000155, PMID: 36275876 PMC9585534

[31] Peng Y Xu Y Wang M Zhang H Xie J. The nnU-Net based method for. Automatic segmenting fetal brain tissues. Health Inf Sci Syst. (2023) 11:17. doi: 10.1007/s13755-023-00220-3 PMC10043149 36998806

[32] Balaur E O’Toole S Spurling AJ Mann GB Yeo B Harvey K. Colorimetric. Histology using plasmonically active microscope slides. Nature. (2021) 598:65–71. doi: 10.1038/s41586-021-03835-2, PMID: 34616057

[33] Yasaka K Hatano S Mizuki M Okimoto N Kubo T Shibata E. Effects of. Deep learning on radiologists' and radiology residents' performance in identifying esophageal cancer on CT. Br J Radiol. (2023) 96:685. doi: 10.1259/bjr.20220685, PMID: 37000686 PMC10546446

[34] Cao K Xia Y Yao J Han X Lambert L Zhang T. Large-scale pancreatic. cancer detection via non-contrast CT and deep learning. Nat Med. (2023) 29:3033–43. doi: 10.1038/s41591-023-02640-w 37985692 PMC10719100

[35] Sui H Ma R Liu L Gao Y Zhang W Mo Z. Detection of incidental esophageal. Cancers on chest CT by Deep Learning. Front Oncol. (2021) 11:700210. doi: 10.3389/fonc.2021.700210 34604036 PMC8481957

[36] Takeuchi M Seto T Hashimoto M Ichihara N Morimoto Y Kawakubo H. Performance of a deep learning-based identification system for esophageal cancer from CT images. Esophagus. (2021) 18:612–20. doi: 10.1007/s10388-021-00826-0, PMID: 33635412

