# Supplementary material for: Esophageal cancer detection via non-contrast CT and deep learning
Source: Front Med (Lausanne). 2024 Mar 6;11:1356752. doi: 10.3389/fmed.2024.1356752 (PMC10953501; doi:10.3389/fmed.2024.1356752)
Supplement: Supplementary file 1 [file Presentation_1.PPTX]

## Slide 1
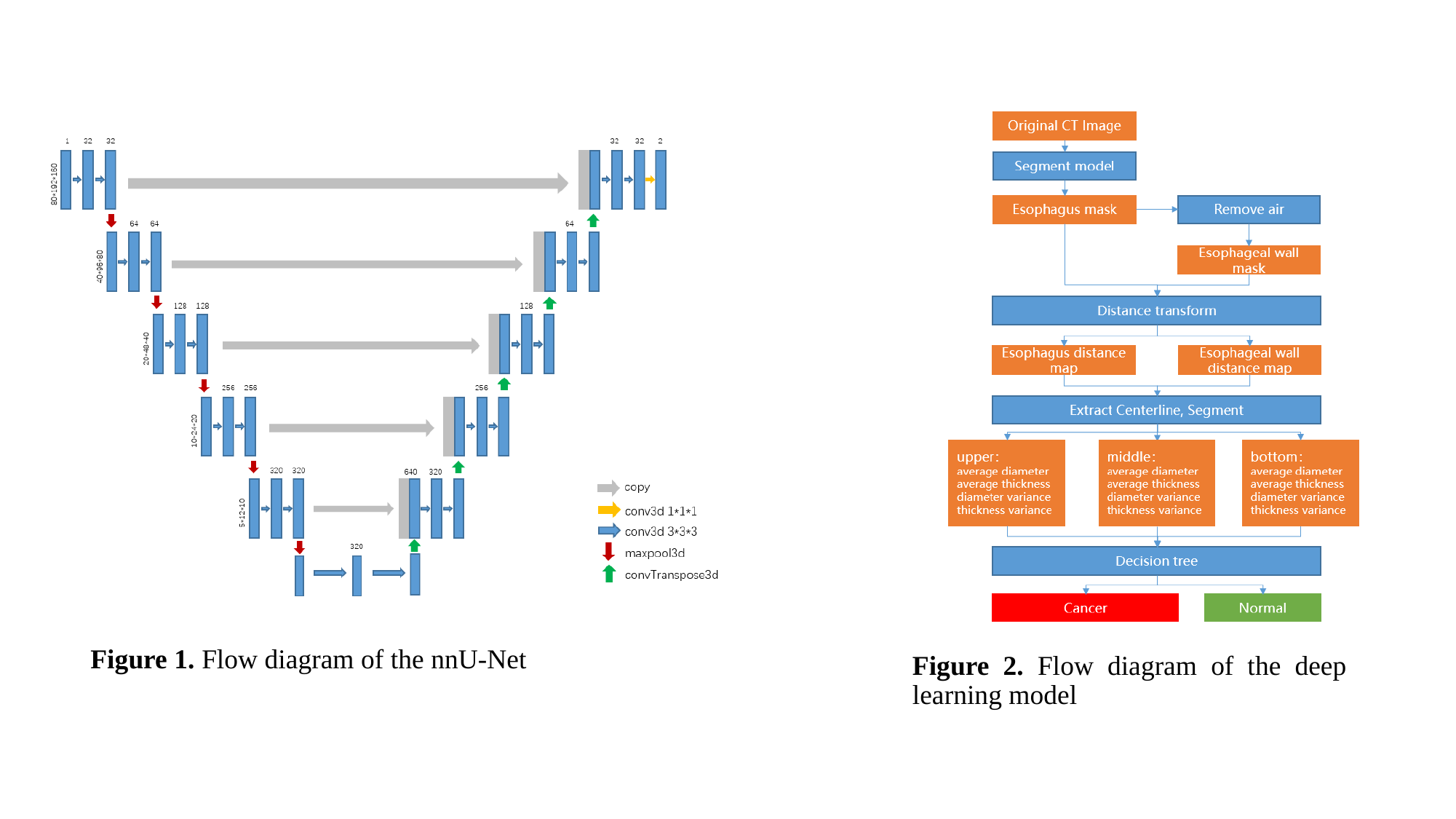

# Figure 1. Flow diagram of the nnU-Net
Figure 2. Flow diagram of the deep learning model

## Slide 2
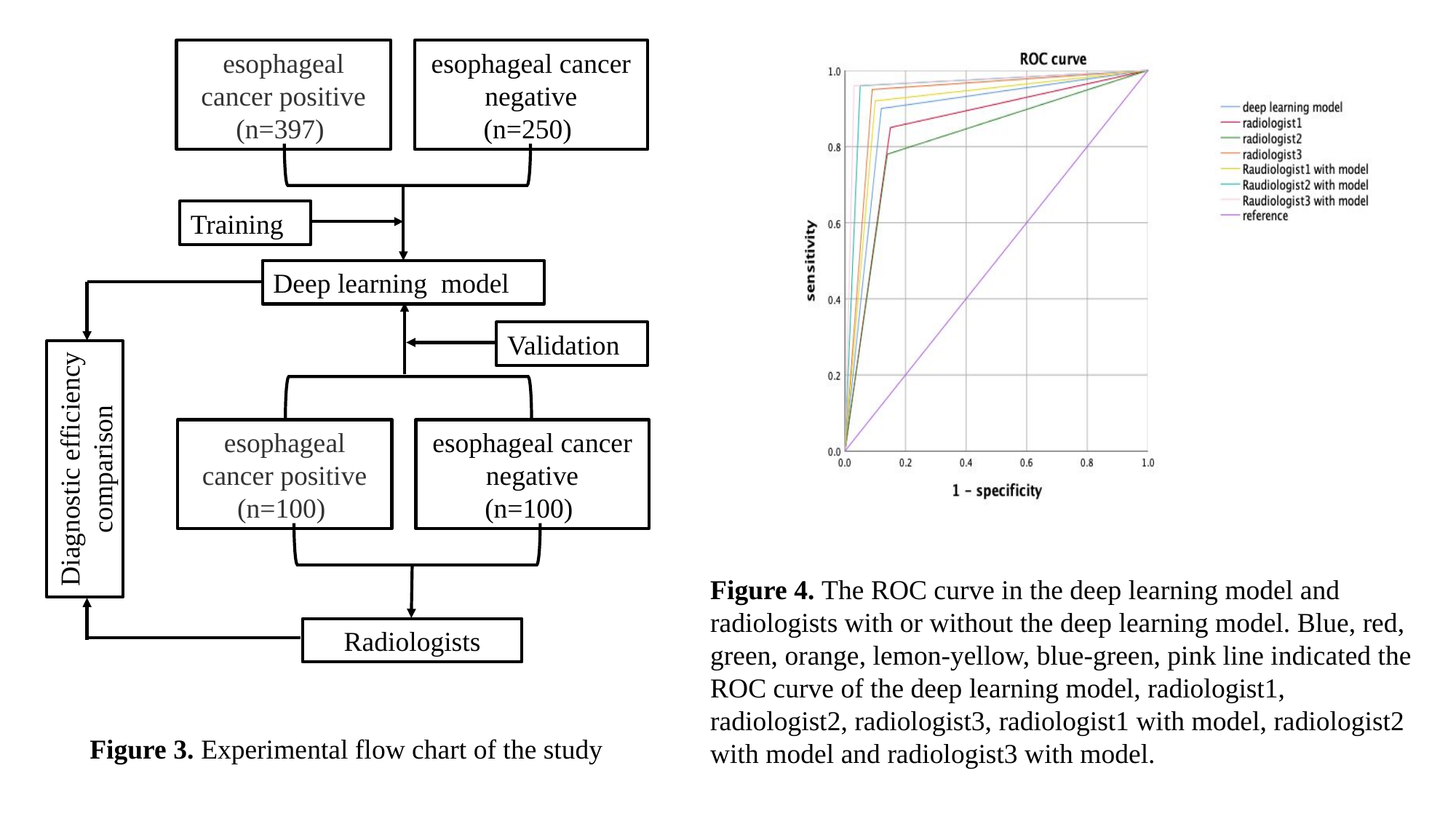

esophageal cancer positive
(n=397)
esophageal cancer negative
(n=250)
Training
Deep learning model
Validation
esophageal cancer positive
(n=100)
esophageal cancer negative
(n=100)
Diagnostic efficiency
comparison
Radiologists
Figure 4. The ROC curve in the deep learning model and radiologists with or without the deep learning model. Blue, red, green, orange, lemon-yellow, blue-green, pink line indicated the ROC curve of the deep learning model, radiologist1, radiologist2, radiologist3, radiologist1 with model, radiologist2 with model and radiologist3 with model.
Figure 3. Experimental flow chart of the study

## Slide 3
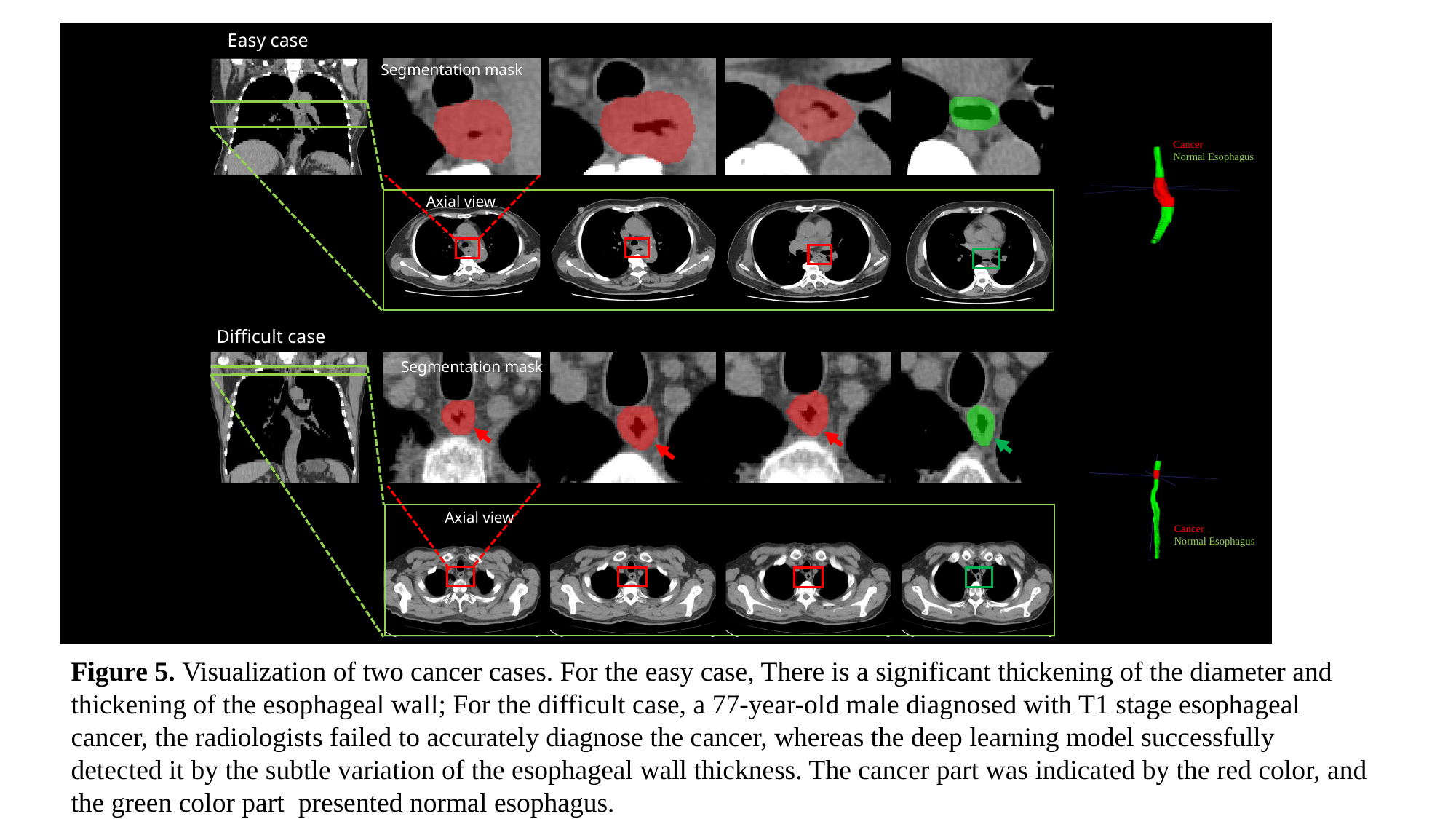

Segmentation mask
Cancer
Normal Esophagus
Axial view
Difficult case
Easy case
Segmentation mask
Axial view
Cancer
Normal Esophagus
Figure 5. Visualization of two cancer cases. For the easy case, There is a significant thickening of the diameter and thickening of the esophageal wall; For the difficult case, a 77-year-old male diagnosed with T1 stage esophageal cancer, the radiologists failed to accurately diagnose the cancer, whereas the deep learning model successfully detected it by the subtle variation of the esophageal wall thickness. The cancer part was indicated by the red color, and the green color part presented normal esophagus.

## Slide 4
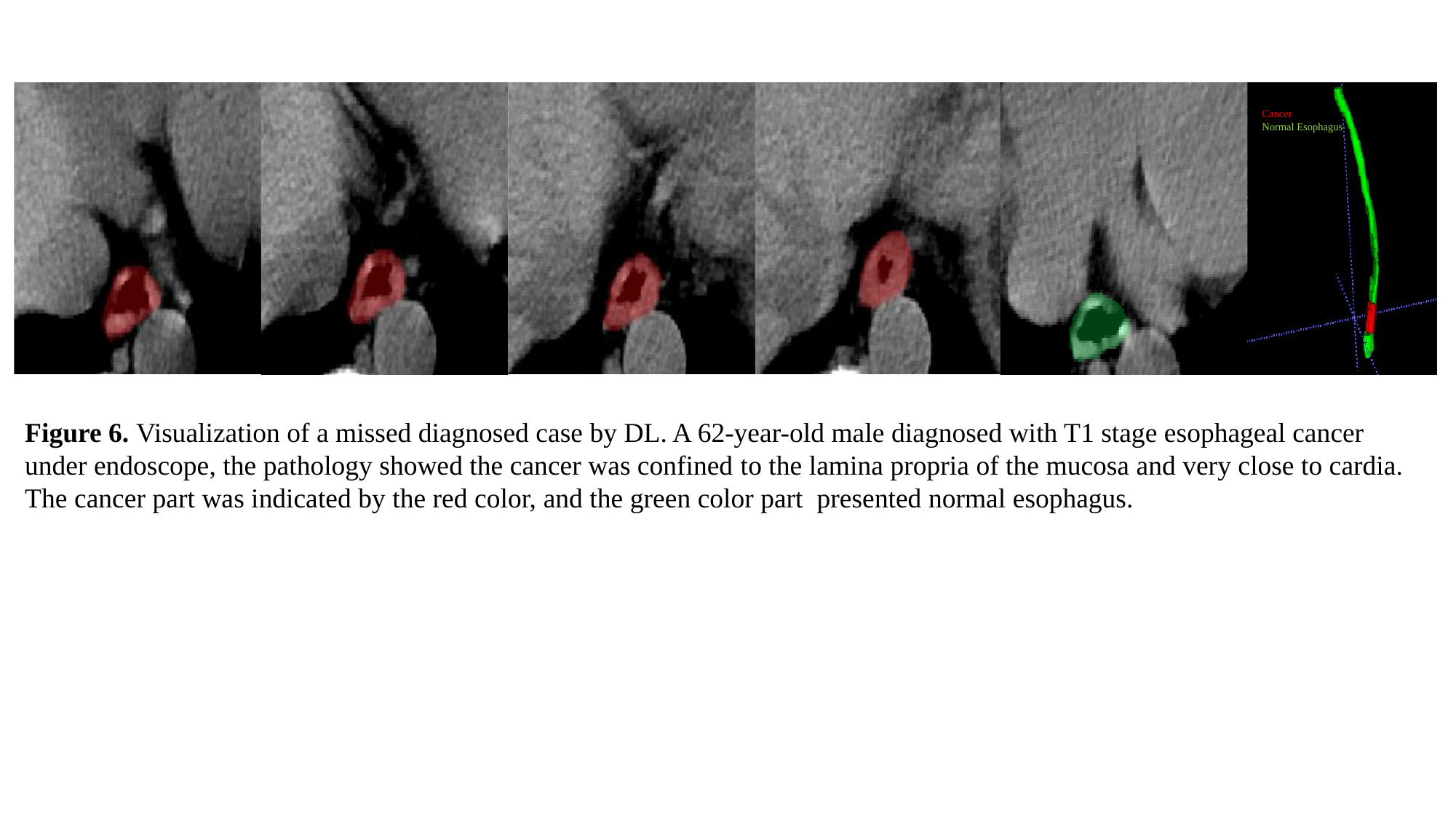

Cancer
Normal Esophagus
Figure 6. Visualization of a missed diagnosed case by DL. A 62-year-old male diagnosed with T1 stage esophageal cancer under endoscope, the pathology showed the cancer was confined to the lamina propria of the mucosa and very close to cardia. The cancer part was indicated by the red color, and the green color part presented normal esophagus.
